# Editorial: Sepsis: studying the immune system to highlight biomarkers for diagnosis, prognosis and personalized treatments

**DOI:** 10.3389/fimmu.2023.1325020

**Published:** 2023-11-22

**Authors:** Juan C. Cutrin, José C. Alves-Filho, Bernhard Ryffel

**Affiliations:** ^1^ Molecular Biotechnology Center II “Guido Tarone”, Department of Molecular Biotechnologies and Sciences for the Health, University of Torino, Turin, Italy; ^2^ Department of Pharmacology, Ribeirão Preto Medical School, University of São Paulo, São Paulo, Brazil; ^3^ Center for Research in Inflammatory Diseases, Ribeirao Preto Medical School, University of São Paulo, São Paulo, Brazil; ^4^ INEM, CNRS, UMR7355, Orléans, France and Experimental and Molecular Immunology and Neurogenetics, University of Orléans, Orleans, France

**Keywords:** sepsis, immune system, biomarkers, immunosuppression, bioinformatic, diagnosis

Sepsis, defined by The Third International Consensus Definitions for Sepsis and Septic Shock (Sepsis-3), is a life-threatening organ dysfunction caused by a dysregulated host response to infection emphasizing the pivotal role-playing by the innate and adaptive immune response in the development of the clinical syndrome (1).

Due to its complex pathogenesis that involves networks of multiple systems, a marked heterogeneity is observed from patient to patient suffering from sepsis, both in terms of organ dysfunction distribution and its severity (2, 3). Despite advances in organ support and antimicrobial therapy up to 25% of patients still succumb to sepsis. But the situation is even more dramatic in septic shock, with a hospital mortality rate of around 60% (4–6). Therefore, there is a high priority to improve the prevention, recognition, diagnosis and management of sepsis.

Unfortunately, there is no single diagnostic available test that establishes the diagnosis of sepsis or septic shock (7, 8). Clinical criteria, that distinguish sepsis from localized microbial infection is a failing host response with multiple organ dysfunction and potentially septic shock (9). Despite the cited consensus Sepsis-3 (1), new criteria will be needed to predict life-threatening sepsis (8).

There is heterogeneity in patient’s responses that may be related to multiple endophenotypes of sepsis that may have implications for personalized treatments (10, 11). Currently, emerging research evaluating omics technologies and bioinformatics methods may improve the care of sepsis patients. Using existing datasets of genetic expression of septic patients, artificial intelligence systems (AI) are trained to recognize disease progression and clinical outcomes (12, 13). For instance, in 2019 Seymour et al. published a paper describing the application of machine learning algorithms to readily available clinical data. Based on the analysis of 29-sepsis-related variables four novel sepsis phenotypes (α, β, γ and δ) with different demographics, laboratory values and patterns of organ dysfunction could be individualized. Compared to other phenotypes, the δ-phenotype is characterized by greater rates of acute renal, hepatic and endothelial dysfunction and mortality rates (14, 15). The pathophysiology of sepsis has recently been reviewed (3, 16, 17).

A major research effort to identify biomarkers in patients with sepsis or septic shock predict mortality has been made. 250 biomarkers have been identified and evaluated including complement system, cytokines, chemokines, cell membrane receptors, soluble receptors, metabolites, damage-associated molecular patterns, non-coding RNAs, miRNAs and more, but no single biomarker or combinations accurately differentiated between and sepsis-like syndrome. Thus, prediction of patient outcomes in sepsis is still driven by clinical signs. It is expected that integrated biomarker-guided algorithms together with the application of AI using machine learning methods may hold promise to improve both the diagnosis and prognosis of sepsis, as well as, to optimize more personalized patient care at the bedside (6, 11, 15, 18).

Moreover, extensive clinical and experimental research has compellingly demonstrated that sepsis can induce a state of immunosuppression, leading to heightened vulnerability to secondary, predominantly opportunistic infections. The mechanisms underpinning sepsis-induced immunosuppression encompass immune cell apoptosis, the proliferation of regulatory T (Treg) cells, and impaired microbial clearance by macrophages (19). In this context, our recent work has yielded fresh insights into the purinergic signaling pathway in severe experimental sepsis in mice. Our findings unveiled a notable expansion of the CD39+ plasmablast population, a key contributor to immunosuppression. This expansion is mediated through adenosine, which inhibits macrophage antimicrobial activity, further accentuating the challenges posed by sepsis-related immunosuppression (20).

In this Research Topic, the authors have provided contributions about crucial aspects of sepsis: (1) pathogenic mechanisms associated with immune dysregulation, (2) identification of biomarkers applicable for diagnosis, and (3) new avenues to accelerate improved treatments for sepsis.

Yao et al. summarized the mechanism of sepsis-associated immunosuppression at the cellular level and highlighted the recent advances in immune monitoring approaches targeting the functional status of both innate and adaptive immune responses. The authors pointed out that strengthening the translational medicine research and application of multi-omics methodologies may provide new insights into the molecular and cellular basis of sepsis-induced immune paralysis and facilitate the identification of novel yet feasible immune-relevant cell-type-specific disease signatures.

Chen et al. employing integrated bulk and single-cell RNA-seq data explored the potential mechanisms of the effects of Ulinastatin (UTI) in sepsis patients. Authors found that the enriched biological processes in myeloid-derived suppressor cells observed comparing UTI versus control samples were those associated with suppressed inflammatory responses. Further cell-cell communication patterns such as ANEXIN, progranulin coding gene and RESISTIN were identified to be differentially regulated in UTI versus control groups. This study provided a comprehensive reference map of transcriptional states of sepsis treated with UTI, as well as a general framework for studying UTI-related mechanisms.

Li et al. hinted at the role that ferroptosis plays during sepsis. Employed cluster Profiler package of R to perform the Gene Ontology and Kyoto Encyclopedia of Genes and Genome enrichment analyses of ferroptosis-related differentially expressed genes they showed that ferroptosis-related gene MAPK14 was of prominent value for the early diagnosis of sepsis in children. They also provided insight into the landscape of immune cells and the expression of immune checkpoints in children underlying sepsis.

Luxen et al. provided a comprehensive overview that highlighted the major signal transduction pathways involved in sepsis-induced endothelial cell activation and dysfunction. Within these pathways – NF-kB, Rac1/RhoA GTPases, AP-1, APC/S1P, Angpt/Tie2, and VEGF/VEGFR2 –the authors focused on the role of kinases and phosphatases as potential druggable targets for therapeutic intervention. Authors also discussed animal studies and clinical trials intervening in these pathways, with emphasis on how the involvement of interconnected kinases and phosphatases from sepsis-associated intracellular signaling networks. Despite the potential that kinase (and phosphatase) inhibitor drugs hold as a treatment for sepsis, several challenges need to be overcome before their applicability for sepsis.

Peng et al. performed an integrated network analysis to identify functional genes concurrently involved in critical illnesses across different etiologies (trauma and sepsis derived from community-acquired pneumonia/abdominal source) and explored the shared signaling pathways of these common genes to tentatively unveil the underlying molecular mechanisms. Authors found that several immune-related biological functions were dysregulated in both trauma and sepsis, identified immune-related common genes, profiled the immune cell proportion, and explored the relationships between them. Notably, they identified a list of 14 immune-related genes among which S100P can predict the prognosis of sepsis patients. The diagnostic and prognostic value of the immune-related common genes were also evaluated to address their potential clinical use as novel biomarkers.

Wang et al. summarized the understanding of long noncoding (lnc) RNAs as a potential new avenue in sepsis-related diagnostic markers and therapeutic targets for sepsis treatment. Furthermore, the authors illustrated the role of lncRNAs in sepsis-induced organ dysfunction. Nevertheless, they pointed out that further studies should be carried out to elucidate the exact underlying molecular mechanisms of lncRNAs during the pathological process of sepsis.

Zhu et al. performed a weighted gene co-expression network analysis in order to identify co-expression modules relating to the outcome of sepsis. They identified the hub gene stromal cell-derived factor 4 (SDF4) which was significantly associated with mortality. Then, authors demonstrated that endoplasmic reticulum (ER) stress tended to be more severe in patients’ peripheral blood mononuclear cells with negative outcomes compared to those with positive outcomes and SDF4 was related to this phenomenon. In addition, in the cecal ligation and puncture (CLP) mouse model of sepsis, authors demonstrated that adenovirus mediated SDF4 overexpression was able to attenuate ER stress. In summary, this study indicates that incorporation of SDF4 may improve clinical parameters predictive value for the prognosis of sepsis and decreased expression levels of SDF4 contribute to excessive ER stress, which is associated with worsened outcomes, whereas overexpression of SDF4 attenuated such activation.

Liu et al. evaluated the relationship between the neutrophil-to-lymphocyte ratio (NLR) combined with interleukin (IL)-6 on admission day and the 28-day mortality of septic patients. Authors showed that the levels of NLR and IL-6 were significantly higher in the deceased patients with sepsis. Although NLR and IL-6 appeared to be independent biomarkers predictor of 28-day mortality in septic patients, the authors suggest that combining NLR with IL-6 might potentially enhance the ability to predict the death risk of patients with sepsis.

Bu et al. using untargeted liquid chromatography-mass spectrometry metabolomics identified and analyzed the common metabolites (acyl carnitine, amino acids, biogenic amines, glycerophospholipids, sphingolipids, and carbohydrates) among patients with sepsis with differences in their 7-day prognosis, as well as the expression of programmed cell death ligand 1 (PD-1) on the surface of CD3+, CD4+, and CD8+ T cells with the scope to analyze the correlation between the differential metabolites and inflammatory factors. They identified three common differential metabolites from the two groups, namely, PC (P-18:0/14:0), 2-ethyl-2-hydroxybutyric acid and glyceraldehyde. These three metabolites were identified as common sepsis metabolites between the 7-day prognosis groups and the PD-1 expression level groups of patients. They may be involved in regulating the expression of PD-1 on the surface of CD4+ T cells through the action of related environmental factors such as IL-2 or lactic acid, which in turn affects the 7-day prognosis of sepsis patients. Authors propose these metabolites as new biomarkers for sepsis diagnostic and prognostic assessments.

In conclusion, there is great progress in understanding of different pathways involved in the complex pathology associated with sepsis. The present knowledge may contribute to monitoring the clinical outcome and guide the clinician in important decisions for patients with severe sepsis. However, there are still huge gaps in critical mechanisms and more clinical and fundamental research will be needed. In summary: the articles provided by the authors show the extensive research which is developing to validate a panel of biomarkers for sepsis diagnosis and prognosis at the bedside. Future studies with the aid of open-source machine learning algorithms will be needed to improve the discovery of circulating biologic markers that may inform sepsis diagnosis and outcomes (21–23).

## Author contributions

JCC: Writing – original draft, Writing – review & editing. JCA-F: Writing – review & editing. BR: Writing – review & editing.
